# Practical cardiovascular risk calculator for asymptomatic patients with type 2 diabetes mellitus: PRECISE‐DM risk score

**DOI:** 10.1002/clc.23405

**Published:** 2020-07-13

**Authors:** Young Choi, Yeoree Yang, Byung‐Hee Hwang, Eun Young Lee, Kun Ho Yoon, Kiyuk Chang, Farouc A. Jaffer, Jae‐Hyoung Cho

**Affiliations:** ^1^ Division of Cardiology, Department of Internal Medicine Seoul St. Mary's Hospital, College of Medicine, The Catholic University of Korea Seoul Republic of Korea; ^2^ Division of Endocrinology and Metabolism, Department of Internal Medicine Seoul St. Mary's Hospital, College of Medicine, The Catholic University of Korea Seoul Republic of Korea; ^3^ Division of Cardiology Cardiovascular Research Center, Massachusetts General Hospital, Harvard Medical School Boston Massachusetts USA

**Keywords:** computed tomography, coronary artery disease, diabetes mellitus, score

## Abstract

**Background:**

Obstructive coronary artery disease (OCAD) is a significant predictor of adverse clinical events in asymptomatic patients with type 2 diabetes mellitus (T2DM).

**Hypothesis:**

We sought to develop an easy‐to‐use risk scoring system to predict OCAD and long‐term clinical outcome in asymptomatic patients with T2DM (PRECISE‐DM).

**Methods:**

A total of 2799 asymptomatic patients with T2DM and no prior coronary disease were consecutively enrolled. OCAD was defined as ≥50% coronary artery stenosis on coronary computed tomography angiography (CCTA). A new risk scoring system was developed in 933 patients undergoing CCTA (derivation cohort) and its performance to predict OCAD and major adverse cardiac and cerebrovascular event (MACCE) was compared with other risk estimates. The scoring system was externally validated in 1899 patients not undergoing CCTA (validation cohort).

**Results:**

The PRECISE‐DM scoring system was created using seven variables that were associated with increased risk of OCAD, with scores ranging from 0 to 9. The scoring system predicted presence of OCAD with a C‐statistic of 0.680 and risk of MACCE with a C‐statistic of 0.708. The UKPDS risk engine and the Framingham risk score showed unreliable performance in prediction of OCAD (C‐statistics 0.531 and 0.577, respectively). Calcium score was highly predictive for OCAD (C‐statistic 0.825) but showed only modest accuracy in predicting MACCE (C‐statistic 0.675). In the external validation cohort, the PRECISE‐DM score showed acceptable discrimination for prediction of MACCE (C‐statistic 0.707).

**Conclusions:**

The PRECISE‐DM scoring system accurately predicted presence of OCAD and risk of MACCE in asymptomatic patients with T2DM.

## 1 INTRODUCTION

Despite advancement in medical treatment options including antiplatelet agents and statins, coronary artery disease (CAD) is still a significant threat to patients with diabetes in terms of morbidity and mortality.^1^ Accurate prediction and early detection of obstructive CAD in asymptomatic patients are particularly important because CAD often progresses without symptoms in diabetic patients. The prevalence of silent significant CAD in diabetic patients was 22% to 33% in studies using myocardial perfusion imaging,^2^, ^3^ and was up to 50% in an autopsy study.^4^

Multi‐slice coronary computed tomography angiography (CCTA) provides accurate non‐invasive imaging of the extent and severity of CAD. Among asymptomatic patients with type 2 diabetes mellitus (T2DM), CCTA detected obstructive CAD in 40% of the subjects.^5^ The presence of obstructive CAD on CCTA also showed a significant correlation with future cardiovascular events.^5^, ^6^ However, the FACTOR‐64 randomized trial found that routine indiscriminate screening by CCTA in asymptomatic patients with T2DM failed to improve clinical outcome.^7^ Considering the low prevalence of severe coronary stenosis (overall 10.6%) and cardiac death rate (1.5%) reported in the FACTOR‐64 study, intensive diagnostic or therapeutic approach would be only beneficial in selective patients at high risk of obstructive CAD in an asymptomatic diabetic population.

In real world practice, a reliable risk prediction model using only clinical variables may confer higher cost‐benefit in identifying individuals who need early intervention for cardiovascular disease. However, the well‐known cardiovascular risk prediction models in the general population, such as the Framingham risk estimate^8^ or DECODE,^9^ gave an unreliable performance in asymptomatic diabetic patients with a greater than 30% underestimation of CAD risk.^10^ The UKPDS risk engine was developed as a more diabetes‐specific risk prediction model for CAD.^11^ However, it showed only modest accuracy in predicting coronary heart disease events in external validation studies and has a significant disadvantage as a practical usage tool because of the complex computation process.^12^, ^13^

This study aimed to develop a new scoring system for **PRE**dicting obstructive **C**oronary artery d**ISE**ase and long‐term clinical outcome in asymptomatic **D**iabetes **M**ellitus (PRECISE‐DM) to provide a reliable, simple‐to‐calculate risk estimate using only clinical variables. The PRECISE‐DM scoring system was created in a prospective cohort of patients with T2DM and without history of ischemic heart disease who underwent CCTA and we externally validated it in a validation cohort not undergoing CCTA.

## 2 MATERIALS AND METHODS

### 2.1 Internal derivation cohort

This was an observational study based on the Coronary CT Angiography Evaluation for Clinical Outcomes in Asymptomatic Patients with type 2 Diabetes Mellitus (CRONOS‐ADM) registry (registered to ClinicalTrials.gov, NCT02070926). The detailed design of the CRONOS‐ADM registry has been described elsewhere.^14^ In brief, asymptomatic patients with T2DM who were > 30 years were enrolled and underwent CCTA. Exclusion criteria were as follows: diagnosis of type 1 diabetes; prior angina or angina‐equivalent symptoms by the Rose questionnaire^15^; use of anti‐anginal medication; history of myocardial infarction (MI), coronary revascularization, cardiac transplantation, life‐threatening condition, or contraindications for use of iodinated contrast media (estimated glomerular filtration rate < 30 mL/min/1.73 m^2^).

### 2.2 External validation cohort

Within 3 months of the primary enrollment, two age‐ and sex‐matched patients per enrolled patient in the derivation cohort were enrolled as an external validation cohort. The inclusion and exclusion criteria were same as the derivation cohort but CCTA was not performed in patients enrolled in the validation cohort. This study was approved by the institutional review board of the Seoul St. Mary's Hospital and performed in accordance with Strengthening the Reporting of Observational Studies in Epidemiology guidelines.^16^ The written informed consent from the patients was waived by the institutional review board as only anonymized data were accessed and analyzed.

### 2.3 CCTA protocol and analysis

CCTA was performed using either a 64‐slice multidetector computed tomography (MDCT) scanner (Light Speed VCT 64, GE Healthcare, Milwaukee, Wisconsin) or a dual‐source computed tomography (DSCT) scanner (Somatom Definition, Siemens Healthcare, Forchheim, Germany). In each patient, 80 to 110 mL of iodinated contrast agent was injected at a flow rate of 5 mL/s with a scan delay of 7 seconds. In the absence of contraindications, each patient with a heart rate >70 beats per minute received intravenous esmolol 1 hour before the scan, and 0.3 mg sublingual dose of nitroglycerin was administered immediately before the scan. The estimated radiation dose ranged from 5 to 14 mSv. Images were reconstructed immediately after completing the scan and transferred to a computer workstation (MDCT: advantage Windows 4.3; GE Healthcare; DSCT: Syngo Multimodality Workplace, version 2008; Siemens Healthcare) for postprocessing.

All scans were analyzed by two experienced radiologists who were blinded to patient clinical information. In accordance with the guidelines of the Society of Cardiovascular Computed Tomography, coronary segments were visually scored for the presence of coronary plaques using a 16‐segment coronary artery model in an intent‐to‐diagnose manner.^17^ Segments were included in the analysis if the diameter was >1.5 mm. The severity of luminal diameter stenosis was scored as none (0% luminal stenosis), nonobstructive (plaques with a lumen narrowing <50%), or obstructive (plaques with maximum stenosis ≥50%). Obstructive CAD in the diagonal branches, obtuse marginal branches, and posterolateral branches was regarded as part of the corresponding major epicardial coronary artery system. The number of diseased vessels was categorized as one, two, three, or left main (LM) coronary artery vessels. The severity of coronary artery calcification was scored using the method developed by Agatston.^18^

### 2.4 Data collection and outcome analysis

Included patients underwent a structured interview for past medical history, laboratory testing and 12‐lead ECG before the CCTA examination. The diagnosis of T2DM was based on the 2010 criteria of the American Diabetes Association, and was defined as fasting glucose ≥126 mg/dL, HbA1c ≥6.5%, and/or postchallenge glucose ≥200 mg/dL.^19^ Patients with a self‐reported or documented history of T2DM under treatment with oral hypoglycemic agents or insulin were also considered to have diabetes. Chronic kidney disease was defined as estimated glomerular filtration rate <60 mL/min/1.73 m^2^. Abnormal ECG was defined as presence of ST‐segment change, pathologic Q wave, or left ventricular hypertrophy (LVH) on resting ECG. Pathologic Q was diagnosed according to the recent definition^20^ and the Romhilt‐Este's index was used to diagnose LVH.^21^

The primary clinical outcome was a major adverse cardiac and cerebrovascular event (MACCE), which was defined as a composite of cardiac death, nonfatal MI, or stroke. The secondary outcome was all‐cause mortality. All clinical outcomes of interest were confirmed by source documents and were centrally adjudicated by a clinical events committee at Seoul St. Mary's Hospital consisting of an independent group of clinicians. For the validation of complete follow‐up data, information on censored survival data and cause of death (cardiac or noncardiac death) was obtained from the Korean Office of Statistics.

### 2.5 Statistical analyses

Continuous variables are presented as mean ± SD and compared using Student's *t*‐tests. Categorical variables are presented as counts with percentages (%) and compared by the Chi‐square test or Fisher's exact test. We used multiple imputations to replace missing values using fully conditional specification approaches based on all candidate predictors and conducted 20 multiple imputations with 50 resampling replications, creating 1000 full datasets. Multivariate logistic regression analysis was used to adjust the risk of obstructive CAD and to identify independent predictors among baseline variables. All significant variables in univariate analysis were considered candidate predictors for the final multivariate logistic regression model. Continuous variables including age, diabetes duration, and HbA1c were categorized by the cutoff with the best discrimination value in the receiver operating characteristic curve. Eight variables (age, sex, prior stroke, hypertension, diabetes duration, HbA1c, use of clopidogrel, and abnormal ECG) were retained in the multivariate model but use of clopidogrel was excluded to avoid multicollinearity. Performance of the final prediction model was evaluated using area under the curve (AUC) analysis and the Hosmer‐Lemeshow goodness‐of‐fit test. The risk score was calculated by dividing each regression coefficient (*β*) by the smallest regression coefficient from the final model and then rounding that number to the nearest integer. The total risk score was calculated for each patient by summation of the score points. The internal validity of the scoring system was assessed by the simulation study, which was formed with 1000 iterations of random partitioning of the data into training and validation sets (50:50 train/test split). The risk score obtained from the training data was applied to the samples in the validation set and the corresponding risk strata were predicted for each sample. This process was iterated 1000 times and the average prediction rate was calculated.^22^ Survival analysis using Cox regression was used to assess the risk of clinical endpoints. Discrimination values of the prediction model for MACCE and all‐cause death were estimated using the Harrell's overall C‐index.^23^ All analyses were two‐tailed, and *P*‐values<.05 were considered to indicate statistical significance. Statistical analyses were performed using the SAS software, version 9.4 (SAS Institute, Cary, North Carolina).

## 3 RESULTS

### 3.1 Baseline characteristics

A total of 933 patients were enrolled in the derivation cohort and underwent CCTA. Baseline characteristics of the derivation cohort are summarized in Table S1. Mean age was 63.4 (±9.6) and 556 (59.6%) were male. Mean duration of diabetes was 11.7 (±9.2) years. Obstructive CAD was detected by CCTA in 374 (40.1%) patients. Among the baseline variables, older age, male sex, longer duration of diabetes, higher HbA1c level, history of hypertension, and prior stroke were significantly associated with presence of obstructive CAD. There was no difference in BMI, smoking ratio, prevalence of dyslipidemia, or serum cholesterol level between patients with and without obstructive CAD. ECG abnormality was more frequently observed in patients with obstructive CAD (25.4% vs 13.9% in the group with obstructive CAD and without, respectively, *P* < .001). More patients with obstructive CAD were receiving insulin therapy (29.7% vs 17.7%, *P* < .001) and clopidogrel (8.8% vs 1.9%, *P* < .001). The prescription rates of aspirin, beta‐blockers, angiotensin‐converting enzyme inhibitor/angiotensin receptor blockers, and statins did not differ between patients with and without obstructive CAD.

The external validation cohort consisted of 1866 patients who did not undergo CCTA at enrollment. The patients had a shorter duration of diabetes, lower HbA1c level, a lower prevalence of dyslipidemia and a higher prevalence of chronic kidney disease compared to the patients in the derivation cohort (Table S2). The prescription rate of aspirin and statin was lower, and the rate of insulin therapy was higher in the validation cohort.

### 3.2 Development of the prediction model

**FIGURE 1:**
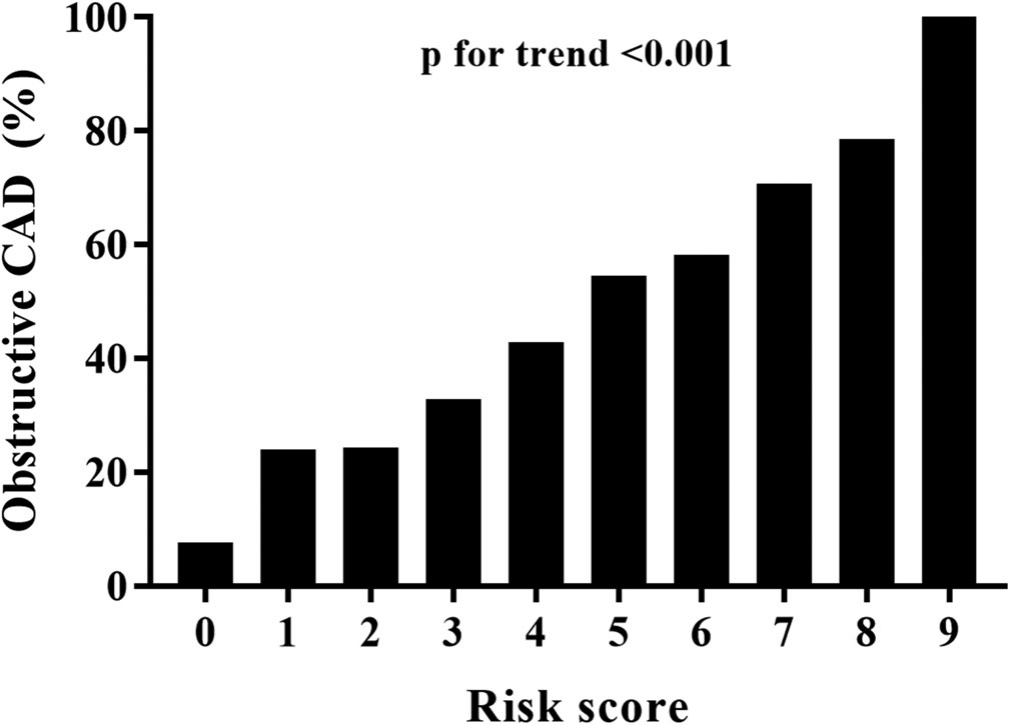
The prevalence of obstructive CAD according to the PRECISE‐DM score. A 1 point increase in the score is associated with approximately 10% increase in the risk of obstructive CAD. CAD, coronary artery disease

Among all significant predictors for presence of obstructive CAD in univariate logistic regression analysis, we found 7 factors (age ≥ 70, male gender, hypertension, prior stroke, diabetes duration ≥10 years, HbA1c ≥7.0, and abnormal ECG) that were associated with increased risk of obstructive CAD in a multivariate analysis (Table 1). Although prior history of stroke was not a significant variable in multivariate analysis, it was included in the final regression model because of the observed trend (*P* = .062) and its established clinical significance. A score prediction model was developed to include the final seven predictors (Table 2). In the scoring system, diabetes duration ≥10 years and abnormal ECG were each assigned two points and other variables were assigned 1 point according to the *β*‐coefficients. The final PRECISE‐DM score model ranged from 0 to 9, and the prevalence of obstructive CAD showed good correlation with the corresponding score (0:7.7%, 1:24.0%, 2:24.3%, 3:32.8%, 4:42.8%, 5:54.6%, 6:58.3%, 7:70.8%, 8:78.6%, 9:100%; Figure 1). A one point increase in the score was associated with approximately 10% higher risk of obstructive CAD, which was statistically significant in the Cochran‐Armitage trend test (*P* < .001).

**TABLE 1:** Univariate and multivariate analyses of the predictors for obstructive CAD

	Univariate analysis			Multivariate analysis		
Variable	HR	95% CI	*P*	HR	95% CI	*P*
Age ≥70	2.03	1.52‐2.21	<.001	1.72	1.23‐2.38	.001
Male	1.46	1.11‐1.91	.006	1.93	1.43‐2.62	<.001
Hypertension	1.56	1.19‐2.03	.001	1.43	1.06‐1.93	.019
Prior stroke	1.95	1.21‐3.14	.006	1.62	0.98‐2.69	.062
DM duration ≥10 years	2.45	1.75‐3.43	<.001	1.86	1.37‐2.53	<.001
Abnormal HDL^a^	1.45	1.05‐1.96	.022	1.37	0.93‐2.02	.108
HbA1c ≥7.0	1.57	1.20‐2.07	.001	1.54	1.13‐2.10	.006
Anemia^b^	1.45	1.05‐2.04	.025	1.06	0.72‐1.55	.756
Use of insulin	1.96	1.44‐2.68	<.001	1.35	0.92‐1.99	.128
Abnormal ECG	2.09	1.50‐2.92	<.001	1.96	1.19‐3.25	.008

Abbreviations: CAD, coronary artery disease; CI, confidence interval; DM, diabetes mellitus, HDL, high‐density lipoprotein; HR, hazard ratio.

^a^ Defined as HDL cholesterol level <60 mg/dL for men and <50 mg/dL for women.

^b^ Defined as hemoglobin level <13 g/dL for men and <12 g/dL for women.

**TABLE 2:** PRECISE‐DM score: predictor variables and assigned scores

Variable	*β*‐coefficient	Assigned score
Age ≥70	.51	1
Male sex	.64	1
Hypertension	.47	1
Stroke	.54	1
HbA1c ≥7.0	.43	1
Abnormal ECG	.67	2
DM duration ≥10 y	.69	2

*Note*: The smallest regression coefficient (.43) of the variable HbA1c ≥7.0 was set as a reference value, and the score of the each variable was assigned according to the ratio of *β*‐coefficients.

Abbreviation: DM, diabetes mellitus.

### 3.3 Validation of the PRECISE‐DM risk score in the internal derivation cohort

The C‐statistic of the PRECISE‐DM risk score was 0.680 (0.632‐0.741), and the score model showed good fitness in the Hosmer‐Lemeshow test (*P* = .339). Using a score cutoff value ≥4, which was determined by a receiver operating characteristic curve with the Youden index, the scoring system predicted a presence of obstructive CAD with 64.2% sensitivity and 63.5% specificity. When a more lenient cutoff value (≥3) was used, the sensitivity was 81.8% and the specificity was 39.7%. In the internal validation analysis, the average prediction rate of the scoring system was 82.0% (±7.6%) in the training set and 82.1% (±7.6%) in the validation set. During a mean follow‐up duration of 37.5 (± 16.5) months, the incidence of MACCE and all‐cause death in the derivation cohort was 33/933 (3.5%) and 42/933 (4.5%), respectively. The PRECISE‐DM risk score showed acceptable discrimination values for prediction of MACCE (C‐statistic 0.708 [0.619‐0.798]) and all‐cause death (C‐statistic 0.672 [0.596‐0.748]).

### 3.4 Validation of the PRECISE‐DM risk score in the external validation cohort

**FIGURE 2:**
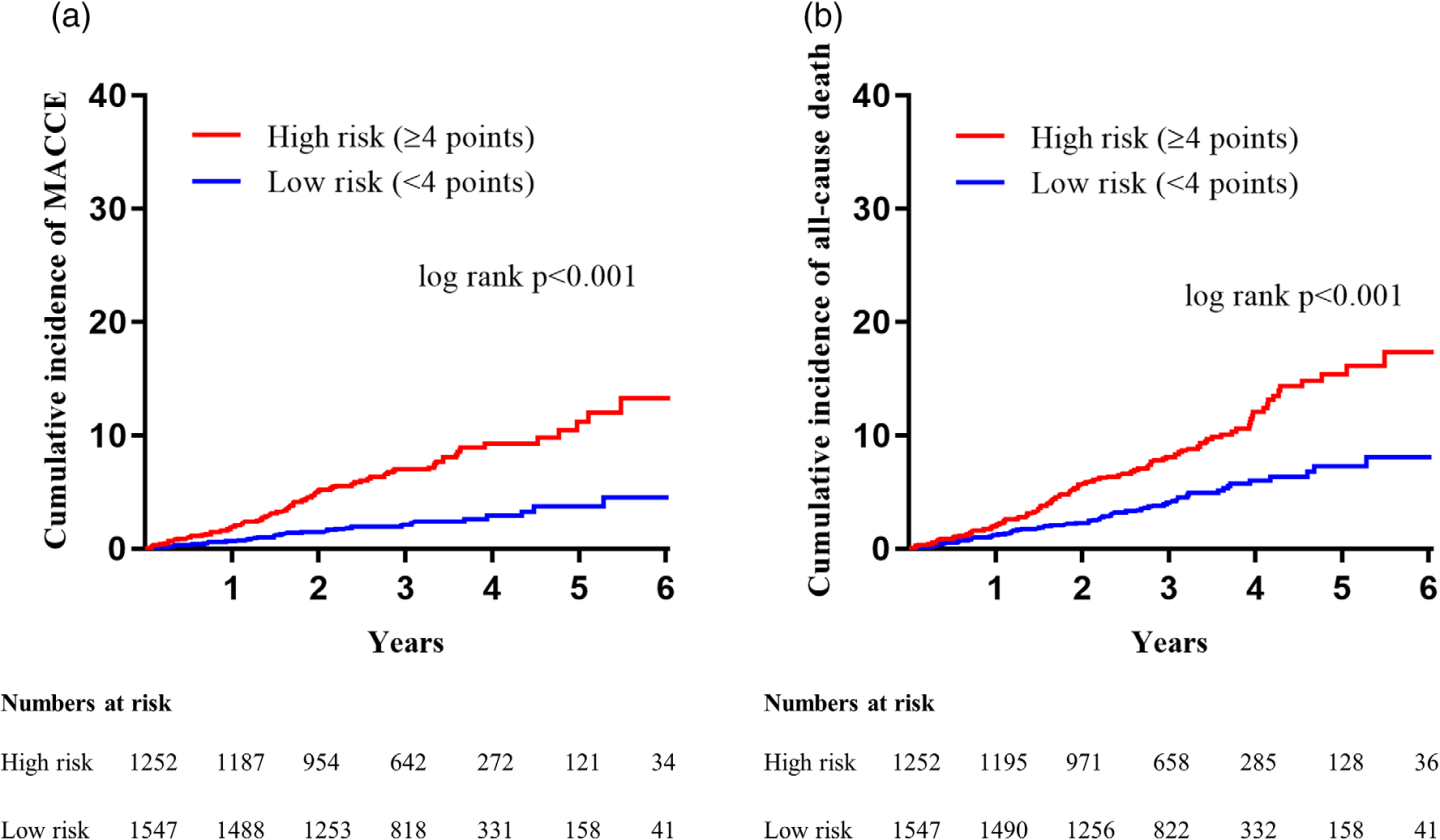
The cumulative incidence of MACCE, A, and all‐cause death, B, in the high risk (PRECISE‐DM score ≥4 points) and the low risk (PRECISE‐DM score <4 points) group in the entire subjects. MACCE, major adverse cardiac and cerebrovascular events

In the validation cohort, MACCE and all‐cause death was observed in 94/1866 (5.0%) and 143/1866 (7.7%) patients, respectively. The PRECISE‐DM score demonstrated C‐statistics of 0.707 (0.655‐0.750) for prediction of MACCE and 0.640 (0.578‐0.702) for prediction of all‐cause death. In the entire population that combined the derivation and validation cohort, the high‐risk group according to the PRECISE‐DM risk score (≥4 points) was associated with 3.2 times higher risk of MACCE (hazard ratio [HR] 3.202, 95% confidence interval [CI] 2.177‐4.710, *P* < .001) and doubled risk of all‐cause mortality (HR 2.094, 95% CI 1.554‐2.281, *P* < .001; Table 3 and Figure 2).

**TABLE 3:** Clinical outcomes according to the risk strata in the entire subjects

	PRECISE‐DM score ≥4 (N = 1252)	PRECISE‐DM score <4 (N = 1547)	HR (95% CI)	*P*
MACCE	91 (7.3%)	36 (2.3%)	3.202 (2.177‐4.710)	<.001
Cardiac death	50 (4.0%)	28 (1.8%)	2.240 (1.410‐3.557)	<.001
Nonfatal MI	14 (1.1%)	2 (0.1%)	8.743 (1.987‐38.470)	.004
Stroke	39 (3.1%)	9 (0.6%)	5.456 (2.643‐11.260)	<.001
All‐cause death	116 (9.3%)	69 (4.5%)	2.094 (1.554‐2.821)	<.001

*Note*: HR and *P*‐value were calculated using univariate Cox‐regression analysis. *P* < .05 indicates statistical significance.

Abbreviations: CI, confidence interval; HR, hazard ratio; MACCE, major adverse cardiac and cerebrovascular event; MI, myocardial infarction.

### 3.5 Comparison between PRECISE‐DM score and other risk predictors

In the internal derivation cohort, the risk of coronary heart disease event calculated by the UKPDS risk estimates was 10.1 (±8.6) %. The C‐statistic of the UKPDS risk estimates was 0.531 for prediction of obstructive CAD and 0.618 for prediction of MACCE in the derivation cohort (Table S3). In the validation cohort, the UKPDS risk estimate showed also lower performance than PRECISE‐DM score in predicting MACCE (C‐statistic 0.653). Mean Framingham risk estimate in the derivation cohort was 21.6 (±8.4) %, and the C‐statistic for prediction of obstructive CAD was 0.577. The C‐statistics of the Framingham risk score for prediction of MACCE was 0.718 in the derivation cohort and 0.633 in the validation cohort. Coronary artery calcium score (CACS) was only available in the derivation cohort, and it was highly predictive of obstructive CAD (C‐statistic of 0.825). However, CACS showed only modest accuracy in predicting MACCE (C‐statistics 0.675).

## 4 DISCUSSION

This new PRECISE‐DM risk score was developed and validated using risk factors associated with obstructive CAD on CCTA and applied to prediction of long‐term clinical outcome in asymptomatic patients with T2DM. This score was intentionally designed to select patients at high‐risk of obstructive CAD among an asymptomatic diabetic population, that may benefit from early diagnostic or therapeutic intervention. The PRECISE‐DM score is an easy‐to‐perform, user‐friendly risk scoring system comprised of seven clinical variables in asymptomatic patients with T2DM. The risk score showed superior discrimination compared to the classical UKPDS risk calculator or the Framingham risk estimates in predicting risk of asymptomatic obstructive CAD. In addition, this scoring system showed acceptable performance in prediction of MACCE in both the internal and external validation datasets. Cutoff score of four best discriminated patients with a high probability of obstructive CAD and worse clinical outcome.

The UKPDS risk engine was used to provide diabetes‐specific risk calculation using eight baseline variables in patients with diabetes and no history of MI or angina. However, external validation studies of the UKPDS risk engine have reported poor calibration and only modest accuracy (C‐statistic 0.61‐0.67) in the prediction of long‐term cardiovascular events.^12^, ^13^, ^24^, ^25^ Other recently introduced diabetes‐specific risk estimate equations including DARTS, Swedish NDR, and the ADVANCE cohort provided improved performance to predict cardiovascular events (C‐statistic 0.69‐0.71).^26^, ^27^, ^28^ However, all of the above risk estimates require 9 to 11 variables and complex equations, which impede their wide adoption in routine clinical practices. Additionally, risk models using clinical endpoints including only cardiac death or acute MI would underestimate the actual prevalence of obstructive CAD, which was also demonstrated in the current study.

The PRECISE‐DM risk score has several advantages over other risk models; (a) Most previous studies did not consider the status of patient symptom for enrollment, whereas we exclusively enrolled patients with no signs or symptoms of CAD. While patients with diabetes and relevant symptoms for CAD are recommended to undergo advanced or invasive cardiac testing, routine screening of CAD is not recommended in asymptomatic diabetic patients.^29^ Thus, risk stratification using clinical variables and identification of those at high risk of obstructive CAD would be mostly helpful in asymptomatic patients with diabetes to apply appropriate diagnostic or therapeutic interventions. (b) The PRECISE‐DM risk score has a far simpler computational system that can be quickly calculated in routine clinical practice. Although simplification of the regression model reduces performance, the PRECISE‐DM risk score still demonstrated acceptable discrimination capacity for detection of obstructive CAD and prediction of MACCE. (c) The PRECISE‐DM score model uses imaging endpoints for definition of CAD, and will be more sensitive to predict the presence of silent obstructive CAD compared to previous models using clinical endpoints. In our study, the Framingham risk calculator and the UKPDS risk estimates resulted in 46 ~ 70% underestimation of prevalence of obstructive CAD. (d) A clinical event is a time‐dependent endpoint, but previous models were validated using AUC based on a logistic regression model. We used Harrell's overall C‐index, which is a better approach to calculate time‐dependent risk estimate in validation of a score system.^23^ (e) The PRECISE‐DM risk score is the first risk model to include ECG variables in the equation. Although resting ECG abnormality is a strong predictor for silent CAD, it has not been included in the previous risk models, which could be partly because precise interpretation of ECG in a large cohort is often difficult.^30^ There is no consensus for the definition of abnormal ECG in prediction of CAD, so we used common abnormal findings associated with myocardial ischemia, including LVH, pathologic Q‐wave, and ST segment abnormalities. The abnormal ECG defined in our cohort was a strong independent predictor for obstructive CAD (HR 1.96, *P* = .008).

Newby et al reported that addition of CCTA to standard care in patients with suspected angina resulted in a 40% reduction of the risk of cardiovascular death or MI in a randomized study.^31^ The benefit in the CCTA group was achieved without greater use of invasive coronary revascularization, and may have been due to higher motivation of the patients and appropriate medical treatment. However, routine CCTA screening in asymptomatic patients would not be cost‐effective, because of the lower probability of obstructive CAD or future cardiovascular events in this population. A simplified risk score can provide prognostic information more conveniently; in our study, the UKPDS risk estimates and the Framingham risk score were not suitable for prediction of OCAD, and although CACS showed higher discrimination value for obstructive CAD, the PRECISE‐DM score was more accurate in prediction of clinical events. Our new score model showed consistent and reliable performance in prediction of obstructive CAD and clinical events, thereby it could be applied for selection of patients to undergo screening with CCTA to improve cardiovascular outcomes.

### 4.1 Limitations

First, the sample size of our study is modest compared to the previous studies for risk model development, mainly because of exclusive inclusion of asymptomatic diabetic patients undergoing CCTA. Second, patients with moderate to severe chronic kidney disease in whom the probability of obstructive CAD is high were excluded due to requirement of contrast agent administration for CCTA.

## 5 CONCLUSION

We developed and validated the PRECISE‐DM risk score as a straightforward and practical clinical scoring system for predicting both a presence of obstructive CAD and long‐term clinical outcome using seven predictors that are routinely available in clinical practice in asymptomatic patients with T2DM. This scoring system would provide risk estimates of obstructive CAD and help identify patients at higher risk of cardiovascular complications with better convenience and performance than previous risk predictors.

## CONFLICT OF INTEREST

The authors declare that they have no conflict of interest and there is no relationship with industry.

## Supporting information


**Table S1** Baseline characteristics according to the presence of obstructive CAD in the derivation cohort.
**Table S2**. Baseline characteristics in the derivation cohort and the validation cohort
**Table S3**. Performances of different risk predictors in the derivation cohort and validation cohort
