# Supplementary material for: Practical cardiovascular risk calculator for asymptomatic patients with type 2 diabetes mellitus: PRECISE‐DM risk score
Source: Clin Cardiol. 2020 Jul 13;43(9):1040–7. doi: 10.1002/clc.23405 (PMC7462187; doi:10.1002/clc.23405)
Supplement: Supplementary file 1 — Table S1 Baseline characteristics according to the presence of obstructive CAD in the derivation cohort. Table S2. Baseline characteristics in the derivation cohort and the validation cohort Table S3. Performances of different risk predictors in the derivation cohort and validation cohort [file CLC-43-1040-s001.docx]

Supplemental table 1. Baseline characteristics according to the presence of obstructive CAD in the derivation cohort.

|  | Obstructive CAD (N=374) | No obstructive CAD (N=559) | p-value* |
| --- | --- | --- | --- |
| Age, years | 65.3±9.5 | 62.1 ± 9.5 | <0.001 |
| Male, n (%) | 243 (64.9%) | 313 (55.9%) | 0.006 |
| Body mass index, kg/m^2^ | 24.2 ± 3.1 | 24.6 ± 3.3 | 0.176 |
| Current smoking, n (%) | 67 (17.9%) | 72 (12.9%) | 0.174 |
| Diabetes duration, years | 14.4 ± 9.9 | 9.9 ± 8.3 | <0.001 |
| SBP, mmHg | 127 ± 14 | 124 ± 14 | 0.003 |
| DBP, mmHg | 75 ± 10 | 76 ± 10 | 0.223 |
| Microangiopathy†, n (%) | 138 (36.9%) | 192 (34.3%) | 0.544 |
| Comorbidities, n (%) |  |  |  |
| Hypertension | 228 (60.9%) | 280 (50.1%) | 0.001 |
| Dyslipidemia | 222 (59.3%) | 336 (60.1%) | 0.906 |
| Prior stroke | 41 (10.9%) | 33 (5.9%) | 0.006 |
| Chronic kidney disease | 20 (5.3%) | 23 (4.1%) | 0.475 |
| Laboratory findings |  |  |  |
| Hemoglobin, g/dL | 13.5 ± 1.7 | 13.6 ± 1.6 | 0.297 |
| Creatinine, mg/dL | 0.90 ± 0.20 | 0.88 ± 0.20 | 0.397 |
| HbA1c, % | 8.1 ± 2.0 | 7.9 ± 1.9 | 0.015 |
| Total cholesterol, mg/dL | 169.4 ± 38.4 | 168.9 ± 37.7 | 0.910 |
| Triglyceride, mg/dL | 134.4 ± 85.1 | 139.7 ± 101.5 | 0.868 |
| HDL cholesterol, mg/dL | 47.0 ± 11.4 | 47.9 ± 12.2 | 0.355 |
| LDL cholesterol, mg/dL | 95.3 ± 33.9 | 93.0 ± 31.9 | 0.453 |
| Abnormal ECG | 95 (25.4%) | 78 (13.9%) | <0.001 |
| Medications, n (%) |  |  |  |
| Aspirin | 178 (47.6%) | 246 (44.0%) | 0.258 |
| Clopidogrel | 33 (8.8%) | 11 (1.9%) | <0.001 |
| Insulin therapy | 111 (29.7%) | 99 (17.7%) | <0.001 |
| Beta-blocker | 48 (12.8%) | 53 (9.4%) | 0.106 |
| ACEi/ARB | 192 (51.3%) | 254 (45.4%) | 0.077 |
| Statin | 209 (55.9%) | 293 (52.4%) | 0.298 |

Categorical variables are presented as number (percentages) and continuous variables are presented as mean ± standard deviation. P<0.05 indicated statistical significance

* P-values were calculated for comparisons between patients with and without obstructive CAD.

† Defined as a presence of diabetic retinopathy or microalbuminuria

CAD = coronary artery disease, SBP = systolic blood pressure, DBP = diastolic blood pressure, HDL = high-density lipoprotein, LDL = low-density lipoprotein, ACEi = angiotensin-converting enzyme inhibitor, ARB = angiotensin receptor blocker

Supplemental table 2. Baseline characteristics in the derivation cohort and the validation cohort

|  | Derivation cohort  (N=933) | Validation cohort (N=1866) | p |
| --- | --- | --- | --- |
| Age, years | 63.4 ± 9.6 | 63.4 ± 9.7 | 0.956 |
| Male, n (%) | 556 (59.6%) | 1114 (59.9%) | 0.956 |
| Body mass index, kg/m^2^ | 24.4 ± 3.2 | 24.2 ± 3.4 | 0.144 |
| Current smoking, n (%) | 139 (14.9%) | 244 (15.3%) | 0.175 |
| Diabetes duration, years | 11.7 ± 9.2 | 9.5 ± 8.7 | <0.001 |
| SBP, mmHg | 125 ± 14 | 126 ± 16 | 0.746 |
| DBP, mmHg | 75 ± 9 | 75 ± 10 | 0.180 |
| Comorbidities, n (%) |  |  |  |
| Hypertension | 508 (54.4%) | 947 (50.8%) | 0.067 |
| Dyslipidemia | 558 (59.8%) | 700 (37.6%) | <0.001 |
| Prior stroke | 74 (8.0%) | 151 (8.2%) | 0.876 |
| Chronic kidney disease | 43 (4.6%) | 265 (14.2%) | <0.001 |
| Laboratory findings |  |  |  |
| Hemoglobin, g/dL | 13.5 ± 1.7 | 13.3 ± 1.8 | 0.007 |
| Creatinine, mg/dL | 0.89 ± 0.20 | 0.99 ± 0.49 | <0.001 |
| HbA1c, % | 8.0 ± 1.9 | 7.6 ± 1.8 | 0.009 |
| Total cholesterol, mg/dL | 169.1 ± 38.0 | 167.1 ± 38.5 | 0.316 |
| Triglyceride, mg/dL | 137.6 ± 95.3 | 133.1 ± 95.0 | 0.269 |
| HDL cholesterol, mg/dL | 47.5 ± 11.9 | 48.1 ± 13.8 | 0.549 |
| LDL cholesterol, mg/dL | 94.0 ± 32.7 | 92.4 ± 32.3 | 0.240 |
| Abnormal ECG | 173 (18.5%) | 338 (19.4%) | 0.886 |
| Medications, n (%) |  |  |  |
| Aspirin | 424 (45.4%) | 593 (31.9%) | <0.001 |
| Clopidogrel | 44 (4.7%) | 92 (4.9%) | 0.820 |
| Insulin therapy | 210 (22.5%) | 529 (28.3%) | 0.001 |
| Beta-blocker | 101 (10.8%) | 194 (10.4%) | 0.727 |
| ACEi/ARB | 446 (47.8%) | 866 (46.4%) | 0.486 |
| Statin | 502 (53.8%) | 910 (48.8%) | 0.012 |

Categorical variables are presented as number (percentages) and continuous variables are presented as mean ± standard deviation. P<0.05 indicated statistical significance

SBP = systolic blood pressure, DBP = diastolic blood pressure, HDL = high-density lipoprotein, LDL = low-density lipoprotein, ACEi = angiotensin-converting enzyme inhibitor, ARB = angiotensin receptor blocker

Supplemental table 3. Performances of different risk predictors in the derivation cohort and validation cohort

|  | **Derivation cohort** | | **Validation cohort** | |
| --- | --- | --- | --- | --- |
|  | C-statistics for OCAD prediction | C-statistics for MACCE prediction | | C-statistics for MACCE prediction |
| PRECISE-DM score | 0.680 | 0.708 | | 0.707 |
| UKPDS risk estimate^11)^ | 0.531 | 0.618 | | 0.653 |
| Framingham risk score^8)^ | 0.577 | 0.718 | | 0.633 |
| CACS^18)^ | 0.825 | 0.675 | |  |

OCAD = obstructive coronary artery disease; MACCE = major adverse cardiac and cerebrovascular event; CACS = coronary artery calcium score
